# Routine use of automated FiO_2_ control in Poland: prospective registry and survey

**DOI:** 10.3389/fped.2023.1213310

**Published:** 2023-08-31

**Authors:** M. Wilinska, T. E. Bachman, P. Piwowarczyk, M. Kostuch, J. Tousty, K. Berła, R. Hajdar, M. Skrzypek

**Affiliations:** ^1^ Department of Neonatology, Centre of Postgraduate Medical Education, Warsaw, Poland; ^2^ Department Medical Technology, School of Biomedical Engineering, Czech Technical University in Prague, Kladno, Czech Republic; ^3^ Clinical Department of Neonatology, Independent Public Hospital, Warsaw, Poland; ^4^ Department of Neonatology and Intensive Neonatal Care, Pomeranian Medical University, Szczecin, Poland; ^5^ Department of Neonatology, Complex of Health Care Facilities, Ostrow Wielkopolski, Poland; ^6^ Department of Neonatology, Provincial Specialist Hospital, Biala Podlaska, Poland; ^7^ Department of Biostatistics Medical University of Silesia, School of Health Sciences in Bytom, Bytom, Poland

**Keywords:** neonatal care, oxygen control, automated oxygen control, adoption of technology, routine use

## Abstract

**Objective:**

The performance of automated control of inspired oxygen (A-FiO2) has been confirmed in dozens of studies but reports of routine use are limited. Broadly adopted in Poland, our aim is to share that experience.

**Methods:**

We used a prospectively planned observational study of the performance, general use patterns, unit practices, and problems with A-FiO2, based on a web registry of case reports, complemented by surveys of subjective impressions.

**Results:**

In 2019, a total of 92 A-FiO2 systems were in routine use in 38 centers. Of the 38 centers, 20 had agreed in 2013 to participate in the project. In these centers, A-FiO2 was applied in infants of all weights, but some centers restricted its use to weaning from oxygen and unstable infants. A cohort had reported their experience with each use (5/20 centers, 593 cases). A quarter of those infants were managed with a lower target range and three-quarters with alarms looser than European guidelines for manual SpO_2_ control. The perceived primary advantages of A-FiO2 were as follows: keeping the readings in the target range, reducing exposure to SpO_2_ extremes, reducing risk from nurse distraction, reducing workload, and reducing alarm fatigue. Practices did evolve with experience, including implementing changes in the alarm strategy, indications for use, and target range. The potential for over-reliance on automation was cited as a risk. There were a few reports of limited effectiveness (moderate 12/593 and poor 2/593).

**Conclusions:**

Automated oxygen control is broadly perceived by users as an improvement in controlling SpO_2_ with infrequent problems.

## Background

Manual titration of inspired oxygen (FiO_2_) is challenging in the newborn ICU. As a result of respiratory instability and frequent desaturations, neonates only spend about half the time in the intended oxygen saturation (SpO_2_) target range (1). Automated control of the FiO_2_ of infants receiving respiratory support (A-FiO2) is now available as an option for most neonatal ventilators in Europe. Numerous studies have consistently reported improved maintenance of the target range and a decrease at high and low SpO_2_ extremes (2, 3). While available in Europe on some ventilators for nearly a decade, its adoption has been slow because of the need to purchase completely new ventilator systems. Widespread adoption of expensive technology is always slow, especially without demonstrably improved outcomes. A large European randomized control trial is underway to determine to what degree this better control results in improved outcomes in extremely low gestational age neonates, which could accelerate adoption if the results are robust (4). Other factors such as investment and access to hands-on experience are also common constraints (5).

However, because Poland was in the midst of updating their neonatal ventilator fleets a decade ago, it had the opportunity to adopt this new technology and remain the only country to broadly implement A-FiO2 use. In 2019 there were 210 neonatal care units in Poland providing respiratory support. These centers had 1,295 ventilators, 92 of which had A-FiO2 capability.

As we have done with the adoption of other neonatal respiratory technologies (6, 7), our neonatal society developed a web-based case registry in 2013 to document its use (8). The aim of this report is to share our experience with the implementation of A-FiO2 in routine clinical practice.

## Methods

This study is an observational report, based on a web-based registry of case reports and written surveys. With the initial acquisition of A-FiO2 in 2013, 21 Polish NICUs agreed to participate in the registry. One dropped out as they did not proceed with A-FiO2 use.

The case report registry was implemented in 2013 and is described elsewhere (8). In summary, the data set used includes infant demographics, baseline clinical status, respiratory support prior to use of A-FiO2, indication for A-FiO2 use, modes of respiratory support during A-FiO2, settings and duration of A-FiO2, and subjective impressions of A-FiO2 functions. The need for informed consent for the collection and use of patient-specific information in the registry was waived by the requisite bioethics research committee (21 November 2013, CMKP-Bioethics Committee, Warsaw Poland). The collection of case reports began in February 2014 and ended in October 2019. There were regular site visits to audit the source documents.

In late 2019, a plan was developed to provide a comprehensive description of the implementation and performance of A-FiO2 (9). It involved complementing the case-by-case information in the registry with survey information. Two written surveys were developed. Most of the information from the survey reflects forced choice answers; either Yes or No or ratings on a 5-point Likert scale. One survey was an overview and the other was detailed. The overview went to all 20 centers and assessed the adequacy of registry enrollment compliance, impressions of A-FiO2 function, staff acceptance, and current indications for use. The second more detailed survey was sent to those centers with good compliance in the registry. The latter survey included seven areas: benefits of A-FiO2, ideal indications for use, barriers to expanded use, problems with A-FiO2, changes in use associated with experience, initial integration into practice, and current indications for use. This survey information was collected in 2020 with input from nurses and physicians. Responses were reviewed and reconciled remotely concurrently with submission. Site visits were planned but were not practical because of the pandemic. In 2021, the surveys were entered into a database, and some final inconsistencies were reconciled remotely.

Differences among centers were evaluated by ANOVA or chi-square as appropriate. A *p* < 0.05 was considered significant. Statistical tests were conducted with XLSTAT V.19.03 software (Addinsoft, Paris, France).

## Results

In 2019, in total 38 centers were using automated FiO_2_ control (A-FiO2) in Poland. Of the 210 NICUs that provide respiratory support, 50 are considered tertiary. Most of these A-FiO2 systems (31/38) were in tertiary-level centers. There were 92 A-FiO2 units in use in 2019. At that time there were only two types in use: 14 were fabian-PRICO and 78 Avea-CLiO2 (Vyaire Medical, Mettowa USA).

All 20 of the centers completed the initial survey covering their overall experience with A-FiO2. These NICUs admitted a median of 250 infants/year (IQR: 185–363). They provided respiratory support with a median of 3 (IQR 1–4) Infant Flow systems and 12 (IQR: 8–14) mechanical ventilators, 3 (IQR: 1–4) with A-FiO2 systems. The responses are detailed in Tables 1A–C. A majority of the centers used the A-FiO2 regularly for “routine care”. The others were more restrictive in their use; “primarily in unstable infants” and “only in intubated infants”. Only one center limited use to preterm infants. The overall performance of A-FiO2 was rated quite positively, though two centers found it occasionally erratic. The initial training by the distributor was rated positively by most centers, though three centers noted some resistance to adoption by the staff. These three centers also reported they rarely used A-FiO2.

**Table 1A:** Indications for Use (20 centers).

How often is A-FiO2 used	Never ☐☐☐☐☐ Routinely	0/6/3/6/5
all infants receiving respiratory support	Yes ☐☐☐☐☐ No	10/4/3/0/3
all unstable infants receiving respiratory support	Yes ☐☐☐☐☐ No	7/2/5/4/2
only intubated infants	Yes ☐☐☐☐☐ No	9/1/2/2/6
only preterm infants	Yes ☐☐☐☐☐ No	1/2/1/0/16

**Table 1B:** Training & Adoption (20 centers).

Training by distributor	Excellent ☐☐☐☐☐ Insufficient	4/14/1/1/0
Rate of adoption by staff	Quickly ☐☐☐☐☐ Resistant	12/4/1/1/2

**Table 1C:** Performance (20 centers).

Effectiveness of A-FiO2	Excellent ☐☐☐☐☐ Poor	11/8/1/0/0
Effectiveness of Alarms	Excellent ☐☐☐☐☐ Excessive	7/11/1/0/1
A-FiO2 ever erratic	Never ☐☐☐☐☐ Regularly	9/7/2/1/1

There were 662 cases documented in the registry. Enrollment at most centers was sporadic between 2013 and 2019. The 4-year period of February 2014–January 2018 was selected as the most reflective of overall use. The compliance, as self-reported, was markedly different in 5 of the 20 centers. In the survey, these 5 indicated “greater than 90%” reporting of the A-FiO2 cases in all but 2 of 20 of the study years. In contrast, the other 15 centers reported <10% in all but 5 of 60 study years. The 5-center cohort was selected. This included 593 infants. Thus 10% of the cases (69/662) were excluded so as to permit reporting of typical use. These 5 centers registered between 42 and 222 cases. The number of cases ranged between 118 and 175 in each of the years. During this period, at these centers the Avea-CLiO2 was the only A-FiO2 system in use.

**Figure 1:**
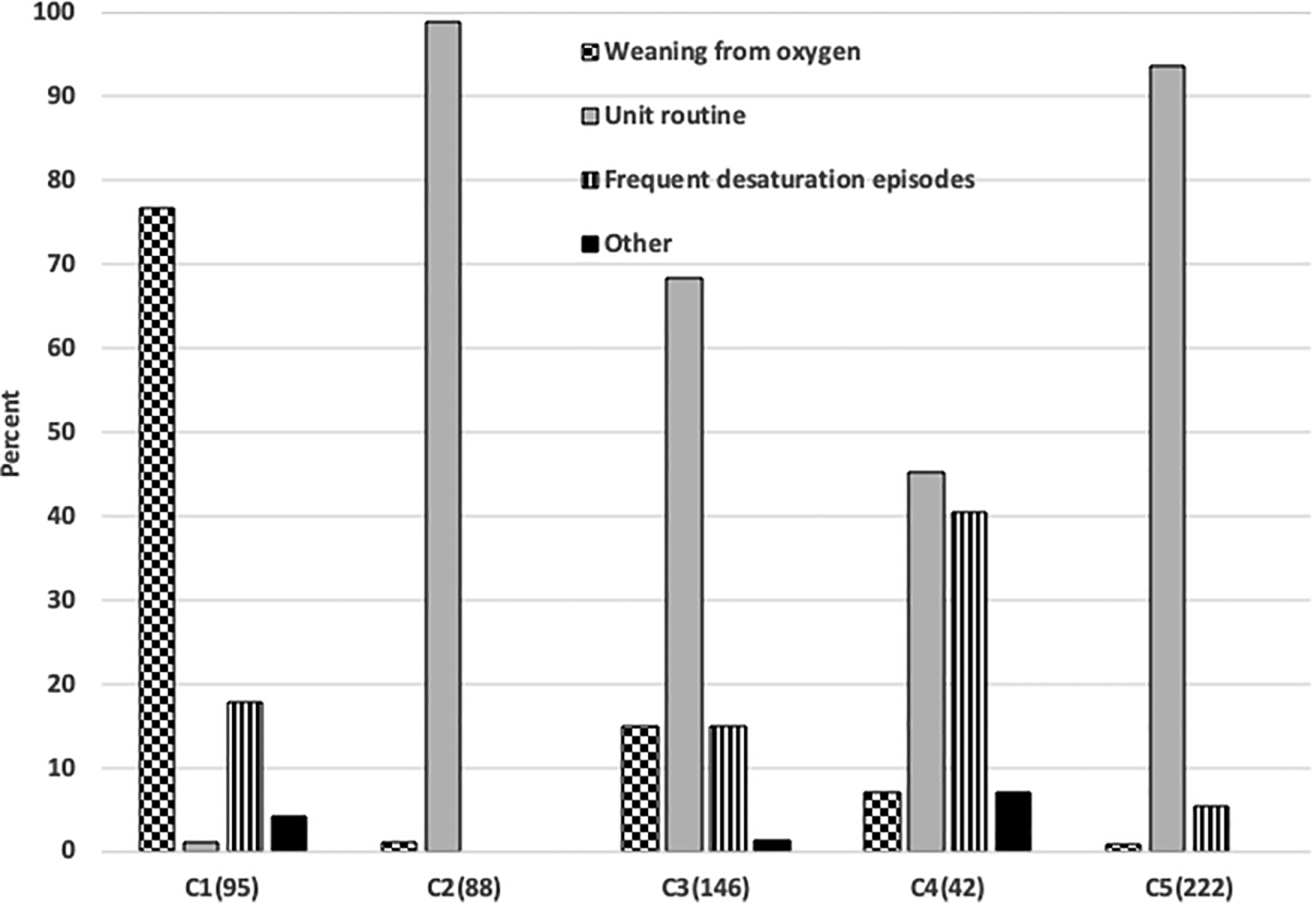
Differences in the indications for Use in 5 centers. The number of cases in each center is shown parenthetically.

The overall indications for use and demographics of these 593 infants are shown in Table 2. The actual indications for use reported varied among the centers (*p* < 0.001), as reflected in Figure 1. Overall, “Routine use” was the primary indication (82%). However, “weaning from oxygen” was the predominate use in one center and “managing infants with frequent desaturations” a common use in another. The limited use in the former seemed to be constrained by the number of A-FiO2 units available, but that was not the case in the latter. Most of the infants were less than 1,500 grams (74%), with a median gestational age of 26 weeks (IQR: 25–28). The larger infants were 2.2 kg (IQR: 1.8–2.9). This was consistent among the 5 centers. This actual case experience is consistent with the overall impressions in the 20-center survey.

**Table 2:** Registry population in 5 centers.

*n*	593
Birth weight (grams)	950 (740–1,525)
Birth gestational age (weeks)	27 (25–31)
Indication for A-FiO2 use (%)	
Unit routine	70
Weaning from oxygen	17
Frequent desaturation episodes	11
Other	2

Median (IQR) or percent proportion.

Details of the actual use of A-FiO2 in the 593 cases are shown in Table 3. A-FiO2 was initiated generally early in the first day of life, with all the centers usually starting in the first 12 h. A-FiO2 was generally utilized for a week or less but use at two centers was several days longer (*p* < 0.001). Most of the infants were intubated when A-FiO2 was initiated and were weaned to noninvasive support during A-FiO2. This was not different among sites. The initial FiO_2_ needed at the initiation of A-FiO2 was moderate but weaned significantly before transitioning from A-FiO2. The infants at the two centers tended to start and end at higher FiO_2_ levels. (*p* < 0.001) This was related to their reported indications of use. The target range was set within 90%–95% SpO_2_ in a majority of infants and set lower in most of the rest. The upper limit of the target range was rarely set higher than 96% (0.7%) but the lower level was occasionally set <88% SpO_2_ (13%). The latter primarily reflects the practice of one center with the typical midpoint of 90%. The target range was only changed during care in 7% of the cases. The alarms were mostly set looser than recommended for manual control (6). They were set with a gap of 1 (as recommended) below in only 20% and above in only 27% of cases. This varied among centers (*p* < 0.001). There were no differences in the set target range or alarm thresholds associated with the two weight categories.

**Table 3:** A-FiO2 management in 5 centers.

*n*	593
Age at initiation of A-FiO2 (hrs)	1.6 (0.2–10.2)
Initial FiO_2_ (%)	40 (30–50)
Initial noninvasive support (%)	17%
Target Range category <90–95, 90–95, >90–95 (%)	22/76/6
Low Alarm gap category <5/5/>5 (%)	44/41/4
High Alarm gap category <3/3/>3 (%)	12/49/39
Duration A-FiO_2_ (days)	3 (1–6)
Final FiO2 (%)	26 (21–41)
Change in FiO2 during A-FiO2	−5 (−19–5)
Final noninvasive support (%)	87%

Median (IQR) or percent proportion.

**Figure 2:**
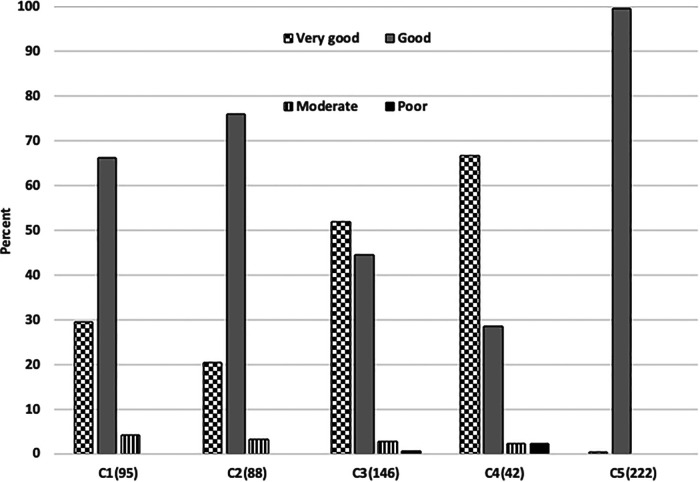
Differences in ratings of performance in 5 centers. The number of cases in each center is shown parenthetically.

Table 4 details the rating of perceived effectiveness of A-FiO2 and alarms in each of the 593 cases. The effectiveness of A-FiO2 was rated overwhelmingly positive. The ratings of effectiveness by center are detailed in Figure 2, with the differences (*p* < 0.001) being primarily between “good” and “very good”. Effectiveness was rated “poor” in only two cases. The question about alarms focused on the perceived frequency of alarms, with the premise that they were set and adjusted during care to achieve the appropriate level of vigilance. The alarms were rarely rated “frequent and persistent”. That rating was associated with the alarms being set tightly compared to loosely (*p* < 0.001).

**Table 4:** A-FiO2 performance in 5 centers.

*n*	593
A-FiO2 effectiveness Rating (%)	
Very good	26
Good	72
Moderate	2
Poor	0.3
Alarm Impression Rating (%)	
Frequent persistent	7
Frequent but not persistent	72
Infrequent	18
Rare	3

percent proportion.

Finally, the five centers also completed a detailed survey of their overall impressions and experiences with training. Tables 5A–C detail the perceived benefits, and problems associated with A-FiO2. All five centers agreed that A-FiO2 improved control of SpO_2_ while reducing the risk of nurse distraction and workload. Furthermore, all but one indicated A-FiO2 reduced alarm fatigue. Less common problems were the differences between the SpO_2_ reading of the A-FiO2 system and the masking of patient deterioration. All but one of the centers felt there was a tendency for the staff to be over-reliant on A-FiO2, a common generic concern for closed-loop control systems. Centers also indicated they did not have enough devices as a barrier to increased utilization of A-FiO2. Resistance to use from clinicians was not a barrier. However, one center indicated limited effectiveness in some infants, and most uses of their A-FiO2 (AVEA-CLiO2) were limited to use with infants who were intubated or receiving nasal IPPV.

**Table 5A:** Perceived benefits in 5 centers.

Better maintenance of SpO_2_ in normoxemia	Yes ☐☐☐☐☐ No	5/0/0/0/0
Reducing episodes of extreme SpO_2_ exposure	Yes ☐☐☐☐☐ No	5/0/0/0/0
Reducing the risk of periodic nurse distraction	Yes ☐☐☐☐☐ No	5/0/0/0/0
Reducing nursing workload	Yes ☐☐☐☐☐ No	5/0/0/0/0
Reducing false/nuisance SpO_2_ alarms	Yes ☐☐☐☐☐ No	4/0/0/1/0

**Table 5B:** Problems with A-FiO2 in 5 centers.

Excessive alarm frequency (alarm fatigue)	No ☐☐☐☐☐ Yes	2/1/0/1/1
Over-reliance on automatic control (ignoring alarms)	No ☐☐☐☐☐ Yes	1/0/0/1/3
A-FiO2 increase of FiO_2_ masked clinical deterioration	No ☐☐☐☐☐ Yes	2/1/0/2/0
Erratic SpO_2_ control	No ☐☐☐☐☐ Yes	4/1/0/0/0
A-FiO2 function stopped	No ☐☐☐☐☐ Yes	5/0/0/0/0
A-FiO2 SpO_2_ reading different from SpO_2_ monitor	No ☐☐☐☐☐ Yes	2/1/1/0/1

**Table 5C:** Why A-FiO2 is not used more in each of 5 centers.

Not enough systems with A-FiO2:	Yes ☐☐☐☐☐ No	3/0/0/2/0
A-FiO2 limited to intubated and NIPPV:	Yes ☐☐☐☐☐ No	4/0/0/0/1
Some clinicians do not prefer the vent with A-FiO2:	Yes ☐☐☐☐☐ No	0/0/0/0/5
Some clinicians do not like A-FiO2:	Yes ☐☐☐☐☐ No	0/0/0/0/5
Clinicians do not like the higher alarm frequency:	Yes ☐☐☐☐☐ No	0/0/1/0/4
A-FiO2 is not always effective:	Yes ☐☐☐☐☐ No	1/0/0/2/2
Lack of adequate training for use of A-FiO2:	Yes ☐☐☐☐☐ No	0/0/0/0/5

Among the five centers, new nurses at the units received 1–4 h of in-service before use, and physicians receive 0.5–2 h. In most centers, the decisions about use were made by the attending physician. This included the decision to use A-FiO2 in all centers and the selection of target range and alarm levels in all but one center, which had a unit standard. The alarm delay settings varied among centers. The usual setting for the centers was 30 s or less but four centers increase them on occasion from the standard level (20–70, 30–120, 15–45, and 8–10 s). As a result of experience with the system two centers reported that they made no changes to their practices. Among the other three centers whose use did evolve with experience, two reported a change in the alarm strategy, two reported a change in the indications for use, and one reported a change in the target range.

## Discussion

Based on a prospective plan, we gathered information about the experience of Polish NICUs during routine use of A-FiO2. This is relevant in that most all the evaluations of A-FiO2 are short cross-over studies of infants <32 weeks gestational age, considerably later in life. Thus, experience with extended use in a typical care environment is wanting. To our knowledge, there is only one other such multicenter report. It is from the UK for 2021, and in it, Kaltsogianni et al. concluded routine use of A-FiO2 was uncommon (10). Pragmatic studies during routine clinical use are limited (11–13) but might also be helpful.

In their report on the adoption of A-FiO2 in the UK, Kaltsogianni et al. found that only 16 of 196 units (8%) used it clinically. This is comparable to what we found in 2019 in level II centers (9/160, 6%). In contrast, most of the tertiary centers in Poland (31/50, 62%) were using A-FiO2. They indicated 11% of the UK centers reported adverse events associated with A-FiO2, with three root causes. These were masking infant deterioration, differences between the control oximeter and the monitor oximeter, and administration of excess oxygen in response to motion artifacts. Anecdotal reports from our centers echo the latter two performance problems, however in our registry, poor performance was rare (2/593 cases). While the adoption was broader in Poland than in the UK, the subjective experiences were similar. Particularly staff belief that A-FiO2 is an improvement in controlling SpO_2_ with infrequent problems and limited staff resistance. Both reports suggest budgetary restraints on the acquisition of new ventilator systems as a primary factor limiting broader use, rather than inconsistent performance or limited indications.

Importantly our study provides some additional information about practices during typical clinical use. This should be useful to centers just implementing A-FiO2 or looking to refine their practices. We found no difference in alarms or target ranges associated with infant maturity. The setting of SpO_2_ alarms is perhaps the most interesting finding. Our study suggests that setting the alarms looser reduced persistent alarms. Looser alarms can be justified in A-FiO2, as they serve a different purpose than during manual control. During manual control, the nurse is alerted that the infant needs attention and response should be prompt to avoid prolonged episodes of hypoxemia or hyperoxemia. During A-FiO2, alarms alert the nurse that the adjustments to FiO_2_ have not addressed the root cause and their attention is needed. However, anecdotal reports suggest some nurses are comfortable with frequent alarms during A-FiO2, as it provides an auditory clue as to the infant’s stability. These looser alarms are outside the European guidelines for preterm infants (14) but those are related to manual FiO_2_ control. One study has demonstrated the effectiveness of a looser alarm strategy in routine practice (13). Except for the practices at one center, most infants were managed with an SpO_2_ target range between 90%–95% consistent with the European guidelines for extremely preterm infants (14). We suggest that A-FiO2 provides the ability to set the target range each day according to the assessment of the infant’s needs, a practice consistent with the AAP guidelines (15), but clearly one not embraced. Finally, other practices relating to in-service for new staff, authority for using A-FiO2, and setting SpO_2_ target ranges and alarm limits and delays should be of interest. While most centers using A-FiO2 in Poland and the UK have good experiences, we would reiterate that initial training and in-service of clinicians new to the department are critical to successful utilization.

There have been about two-dozen controlled trials of A-FiO2 and nearly all have enrolled primarily infants <33 weeks gestational age. However, we reported, consistent with the UK, that it is used clinically on infants of all ages. We reported that 26% were larger than 1,500 grams. Also, in Poland and the UK most centers use it routinely rather than for narrower clinical indications. Of note, the large ongoing outcomes study of A-FiO2 is only enrolling infants with a gestational age of <28 weeks starting within the first 2 days of life (4). This is clearly the group of infants that are most likely to show a marked improvement with better oxygen control. Demonstration of improved outcomes would certainly support increased budgets and drive adoption. Nevertheless, these represent a small portion of neonates on respiratory support where it is routinely used. Also, the risks associated with alarm fatigue are well understood and are highly relevant in the NICU; and we reported A-FiO2 reduced alarm fatigue. Finally, A-FiO2 seems to provide the potential for labor savings and certainly provides an important safety net for episodes with inadequate staffing for acuity.

There are several limitations to our study. First, it is an observational study. While some of the metrics reported are objective, many of the important ones are subjective impressions. Such descriptive reports should be interpreted with caution. Nevertheless, we feel these subjective impressions of functionality, resistant to adoption and standard practices should be helpful in refining or initiating new therapies. There is also some bias in that the impressions no doubt reflect those of the nursing and physician supervisors. Neonatal care differs among countries as do the practices among centers and clinicians. These findings should be evaluated in the context of the reader’s clinical environment. Nevertheless, the stress of nurses, risk of alarm fatigue, and shortage of staff are common problems everywhere. Additionally, the details of our report come from a sample of convenience. Finally, our registry represents the experience of five centers and is too small to establish or evaluate different clusters of practice.

Automated oxygen control is broadly adopted in tertiary care neonatal centers in Poland and used in some other centers. It is perceived by nurses and physicians as an improvement in controlling SpO_2_ with infrequent problems. We hope our experience and practices are useful to those considering implementing A-FiO2 and helpful to those with less experience than Poland. Additional research is warranted to evaluate the impact on staffing, adverse events, and clinical outcomes.

## Data Availability

The raw data supporting the conclusions of this article will be made available by the authors, without undue reservation.
