# Supplementary material for: Routine use of automated FiO2 control in Poland: prospective registry and survey
Source: Front Pediatr. 2023 Aug 31;11:1213310. doi: 10.3389/fped.2023.1213310 (PMC10502171; doi:10.3389/fped.2023.1213310)
Supplement: Supplementary file 1 [file Datasheet1.pdf]

SUPPLEMENT

Written Survey1
This survey was developed in English, then translated to Polish and sent to the centers.

Table 1 Compliance with Registry
Does enrollment reflect actual use ?

2014	Complete (>90%) ☐☐☐☐☐ No <10%)
2015	Complete (>90%) ☐☐☐☐☐ No <10%)
2016	Complete (>90%) ☐☐☐☐☐ No <10%)
2017	Complete (>90%) ☐☐☐☐☐ No <10%)
2018	Complete (>90%) ☐☐☐☐☐ No <10%)
2019	Complete (>90%) ☐☐☐☐☐ No <10%)

For each year, why ?

Table 2 Overall Impression of A-FiO2
2a) Rate current subjective impressions about A-FiO2 use

How often is A-FiO2 used:	Routinely ☐☐☐☐☐ Never
Effectiveness of A-FiO2:	Excellent ☐☐☐☐☐ Poor
Effectiveness of Alarms:	Excellent ☐☐☐☐☐ Excessive
A-FiO2 ever erratic:	Never ☐☐☐☐☐ Regularly
Rate of adoption by staff:	Quickly ☐☐☐☐☐ Resistant
Training by distributor:	Excellent ☐☐☐☐☐ Insufficient

b) Current Indications for use

all infants receiving respiratory support:	Yes ☐☐☐☐☐ No
all unstable infants receiving respiratory support:	Yes ☐☐☐☐☐ No
only intubated infants:	Yes ☐☐☐☐☐ No
only preterm infants:	Yes ☐☐☐☐☐ No

Written Survey 2
This survey was developed in English, then translated to Polish and sent to the centers.

3a) About the benefits of A-FiO2

better maintenance of SpO2 in normoxemia	Yes ☐☐☐☐☐ No
reducing episodes of extreme SpO2 exposure	Yes ☐☐☐☐☐ No
reducing the risk of periodic nurse distraction	Yes ☐☐☐☐☐ No
reducing nursing work load	Yes ☐☐☐☐☐ No
reducing false/nuisance SpO2 alarms	Yes ☐☐☐☐☐ No

3b) About infants whom A-FiO2 should ideally be used?

all infants receiving respiratory support:	Yes ☐☐☐☐☐ No
all unstable infants receiving respiratory support:	Yes ☐☐☐☐☐ No
only intubated infants:	Yes ☐☐☐☐☐ No
only preterm infants:	Yes ☐☐☐☐☐ No

3c) About why A-FiO2 is not used more in this unit ?

not enough systems with A-FiO2:	Yes ☐☐☐☐☐ No
A-FiO2 limited to intubated and NIPPV:	Yes ☐☐☐☐☐ No
some clinicians do not prefer the vent with A-FiO2:	Yes ☐☐☐☐☐ No
some clinicians do not like A-FiO2:	Yes ☐☐☐☐☐ No
clinicians do not like the higher alarm frequency:	Yes ☐☐☐☐☐ No
A-FiO2 is not always effective:	Yes ☐☐☐☐☐ No
lack of adequate training on use of A-FiO2:	Yes ☐☐☐☐☐ No

3d) About problems with A-FiO2

excessive alarm frequency (alarm fatigue)	Yes ☐☐☐☐☐ No
over reliance on automatic control (ignoring alarms)	Yes ☐☐☐☐☐ No
A-FiO2 increase of FiO2 masked clinical deterioration	Yes ☐☐☐☐☐ No
erratic SpO2 control	Yes ☐☐☐☐☐ No
A-FiO2 function stopped	Yes ☐☐☐☐☐ No
A-FiO2 SpO2 reading different from SpO2 monitor	Yes ☐☐☐☐☐ No
Any other problem	Yes ☐☐☐☐☐ No

For any of these not NO, discuss and describe both details and frequency

3e) About changes in your use of A-FiO2 that came with increased experience

Setting level for the control range	Yes ☐☐☐☐☐ No
Setting of the SpO2 alarm thresholds	Yes ☐☐☐☐☐ No
Setting of the SpO2 alarm delay	Yes ☐☐☐☐☐ No
Indications for use (infant size, diagnosis, acuity)	Yes ☐☐☐☐☐ No

For any of these not NO, discuss and describe details including whether it was unit wide or related to individual clinicians.

3f) About the initial integration of A-FiO2 into your unit

Did you receive training from the distributor or manufacture	Yes ☐ No ☐
Was the initial training adequate?	Yes ☐☐☐☐☐ No
Time between training and start of routine use	________ weeks
How long is the orientation for physicians new to A-FiO2	________ hours
How long is the orientation for nurses new to A-FiO2	________ hours

3g) About the <u>current</u> use of A-FiO2 in your unit?

Prescribed target ranges are part of the medical record	Yes ☐ No ☐
Prescribed SpO2 alarm levels are part of the medical record	Yes ☐☐☐☐☐ No
A-FiO2 is turned on or off only by the attending physician	Yes ☐☐☐☐☐ No
Target Ranges are set or reset only by the attending physician	Yes ☐☐☐☐☐ No
Alarm delays are set or reset only by the attending physician	Yes ☐☐☐☐☐ No
SpO2 Alarm delays are usually set at	_______ seconds
SpO2 Alarm delays are on occasion set between	________-________ s

Web-Registry-data fields

The web input pages were all developed in Polish. This Table of the fields was translated to English in the database reports to the primary investigators.

Field	Type	SAS
IdClio	int(10) unsigned	IdClio
refNo	varchar(30)	refNo
delivDate	datetime	delivDate
gender	enum('m','f','u')	gender
weight	int(11)	weight
age	int(11)	age
delivRoute	enum('Cesarean','Vaginal')	delivRoute
gestation	enum('Single','Multiple')	gestation
steroids	enum('NO','YES','No Data')	steroids
resNeopuff	enum('NO','YES')	resNeopuff
resAmbuBag	enum('NO','YES')	resAmbuBag
resNonInvasive	enum('NO','YES')	resNonInvasive

resFiO2Max	int(11)	resFiO2Max
resIntubation	enum('NO','YES')	resIntubation
resCardiacMassage	enum('NO','YES')	resCardiacMassage
resDrugs	enum('NO','YES')	resDrugs
insure	enum('NO','YES')	insure
lisa	enum('NO','YES')	lisa
otherSurfactant	enum('NO','One','Multiple')	otherSurfactant
beforeA-FiO2_NCPAP	enum('NO','YES')	beforeA-FiO2_NCPAP
beforeA-FiO2_NIPPV	enum('NO','YES')	beforeA-FiO2_NIPPV
beforeA-FiO2_MV	enum('NO','YES')	beforeA-FiO2_MV
beforeA-FiO2_HFO	enum('NO','YES')	beforeA-FiO2_HFO
beforeA-FiO2_FiO2Min	int(11)	beforeA-FiO2_FiO2Min
beforeA-FiO2_FiO2Max	int(11)	beforeA-FiO2_FiO2Max
previousFiO2	int(11)	previousFiO2
A-FiO2Date	datetime	A-FiO2Date
FiO2startSetting	int(11)	FiO2startSetting
A-FiO2SpO2Mode	enum('Invasive','NonInvasive')	A-FiO2SpO2Mode
A-FiO2SpO2Date	datetime	A-FiO2SpO2Date
SpO2targetMin	int(11)	SpO2targetMin
SpO2targetMax	int(11)	SpO2targetMax
SpO2targetChanged	enum('NO','YES')	SpO2targetChanged
SpO2alarmMin	int(11)	SpO2alarmMin
SpO2alarmMax	int(11)	SpO2alarmMax
A-FiO2SpO2Reason	enum('Unit routine','Frequent desaturation episodes','Weaning from oxygen','Other')	A-FiO2SpO2Reason
A-FiO2SpO2Opinion	enum('Poor','Moderate','Good','Very good')	A-FiO2SpO2Opinion
A-FiO2SpO2alarmsOpinion	enum('Frequent persistent','Frequent but not persistent','Infrequent','Rare')	A-FiO2SpO2alarmsOpinion
weaningDate	datetime	weaningDate
weaningFiO2	int(11)	weaningFiO2
supportAfterWeaning_Oxygen	enum('NO','YES')	supAfterWean_Oxygen
supportAfterWeaning_NCPAP	enum('NO','YES')	supAfterWean_NCPAP
supportAfterWeaning_NIPPV	enum('NO','YES')	supAfterWean_NIPPV
supportAfterWeaning_otherInvasive	enum('NO','YES')	supAfterWean_otherInvasive
outcomesDay28	enum('Death','At home','Transferred to other hosptial','Still on board')	outcomesDay28
outcomesDischarge	enum('Death','At home','Transferred to other hospital')	outcomesDischarge
dischargeDate	date	dischargeDate
endOfSupportDate	datetime	endOfSupportDate

ROP	enum('0','I','II','III','IV')	ROP
ROPlaser	enum('NO','YES')	ROPlaser
BPD	enum('No','Mild','Moderate','Severe','NoData')	BPD
PDA	enum('NO','YES','NoData')	PDA
PDA_treatment	enum('Medical','Surgical','No treatment')	PDA_treatment
IVH	enum('No','Below III','III','IV','NoData')	IVH
insClio	datetime	insClio
modifClio	datetime	modifClio
modifIdOsoba	varchar(15)	modifIdOsoba
fkIdOsoba	varchar(15)	fkIdOsoba
lockRecord	tinyint(1)	lockRecord
